# Satsurblia: New Insights of Human Response and Survival across the Last Glacial Maximum in the Southern Caucasus

**DOI:** 10.1371/journal.pone.0111271

**Published:** 2014-10-29

**Authors:** Ron Pinhasi, Tengiz Meshveliani, Zinovi Matskevich, Guy Bar-Oz, Lior Weissbrod, Christopher E. Miller, Keith Wilkinson, David Lordkipanidze, Nino Jakeli, Eliso Kvavadze, Thomas F. G. Higham, Anna Belfer-Cohen

**Affiliations:** 1 Earth Institute and School of Archaeology, University College Dublin, Dublin, Ireland; 2 Georgian State Museum, Department of Prehistory, Tbilisi, Georgia; 3 Israel Antiquities Authority, Jerusalem, Israel; 4 Zinman Institute of Archaeology, University of Haifa, Haifa, Israel; 5 Institute for Archaeological Sciences, and Senckenberg Centre for Human Evolution and Paleoenvironment, University of Tübingen, Tübingen, Germany; 6 Department of Archaeology, University of Winchester, Winchester, United Kingdom; 7 Institute of Paleobiology, National Museum of Georgia, Tbilisi, Georgia; 8 Oxford Radiocarbon Accelerator Unit, Research Laboratory for Archaeology & the History of Art, University of Oxford, Oxford, United Kingdom; 9 Institute of Archaeology, Hebrew University, Jerusalem, Israel; Universidade do Algarve, Portugal

## Abstract

The region of western Georgia (Imereti) has been a major geographic corridor for human migrations during the Middle and Upper Palaeolithic (MP/UP). Knowledge of the MP and UP in this region, however, stems mostly from a small number of recent excavations at the sites of Ortvale Klde, Dzudzuana, Bondi, and Kotias Klde. These provide an absolute chronology for the Late MP and MP–UP transition, but only a partial perspective on the nature and timing of UP occupations, and limited data on how human groups in this region responded to the harsh climatic oscillations between 37,000–11,500 years before present. Here we report new UP archaeological sequences from fieldwork in Satsurblia cavein the same region. A series of living surfaces with combustion features, faunal remains, stone and bone tools, and ornaments provide new information about human occupations in this region (a) prior to the Last Glacial Maximum (LGM) at 25.5–24.4 ka cal. BP and (b) after the LGM at 17.9–16.2 ka cal. BP. The latter provides new evidence in the southern Caucasus for human occupation immediately after the LGM. The results of the campaigns in Satsurblia and Dzudzuana suggest that at present the most plausible scenario is one of a hiatus in the occupation of this region during the LGM (between 24.4–17.9 ka cal. BP). Analysis of the living surfaces at Satsurblia offers information about human activities such as the production and utilisation of lithics and bone tools, butchering, cooking and consumption of meat and wild cereals, the utilisation of fibers, and the use of certain woods. Microfaunal and palynological analyses point to fluctuations in the climate with consequent shifts in vegetation and the faunal spectrum not only before and after the LGM, but also during the two millennia following the end of the LGM.

## Introduction

The Southern Caucasus region played a major role in human evolution. During the past two decades research in this region has mainly focused on the Middle-Upper Palaeolithic (MP-UP) transition in the context of Neanderthal extinction and the first appearance of modern humans [1]–[4]. Less emphasis has been placed on charting human occupation during and right after the Last Glacial Maximum (LGM) at 24,000–18,000 calibrated years before present (ka cal. BP), a climate event which had a major demographic impact on human populations in Eurasia [5]–[6].

**Figure 1:**
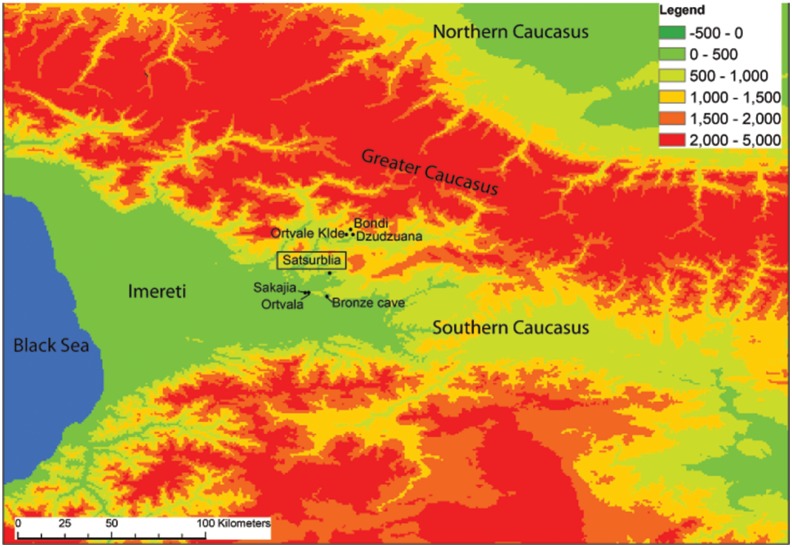
Location of Satsurblia Cave and other key sites with Upper Palaeolithic occupation in western Georgia.

The interdisciplinary project reported on here focuses on fieldwork at Satsurblia cave, western Georgia (Fig. 1), a site discovered in 1975 by A. N. Kalandadze [7], and who subsequently excavated it sporadically during 1976, 1985–88. Excavations were also carried out by K. Kalandadze in 1989–1992 [8], and by T. Meshveliani in 2008–2010.

Before the start of our campaigns current knowledge about UP occupation in Western Georgia was based predominantly on the study of Dzudzuana cave which has a UP archaeological sequence comprising three occupational episodes separated by millennia-long hiatuses: the lowermost UP phase, Unit D, dated to 34.5–32.2 ka cal. BP; followed by Unit C, dated to 27–24 ka cal. BP; and the latest UP phase, Unit B dated to 16.5–13.2 ka cal. BP [2]. It is unclear, however, whether the hiatuses are due to the absence of humans in the entire region or rather if they reflect the particular depositional and occupational history of this specific site. Analyses of the faunal assemblages from Dzudzuana, Ortvale Klde, and Kotias Klde (the latter two sites yielded short UP sequences) indicate that hunting had focused on a few ungulate species with observed temporal variations in their relative proportions. Since there are limited differences in taphonomic history across the sites, it was postulated that the composition reflects variations in the availability of animal resources by season and period [2], [9]–[11].

In this paper we report on the record from Satsurblia and combine that with data from the sites of Dzudzuana, Ortvale Klde and Kotias Klde in order to develop a new regional Upper Palaeolithic archaeological sequence. This is followed by faunal data which provide information on the subsistence and behavior of humans during this period, and information about local palaeoclimate and palaeoecology in order to study the effect of climatic oscillations on human populations.

## Stratigraphy and Radiocarbon Dating

**Figure 2:**
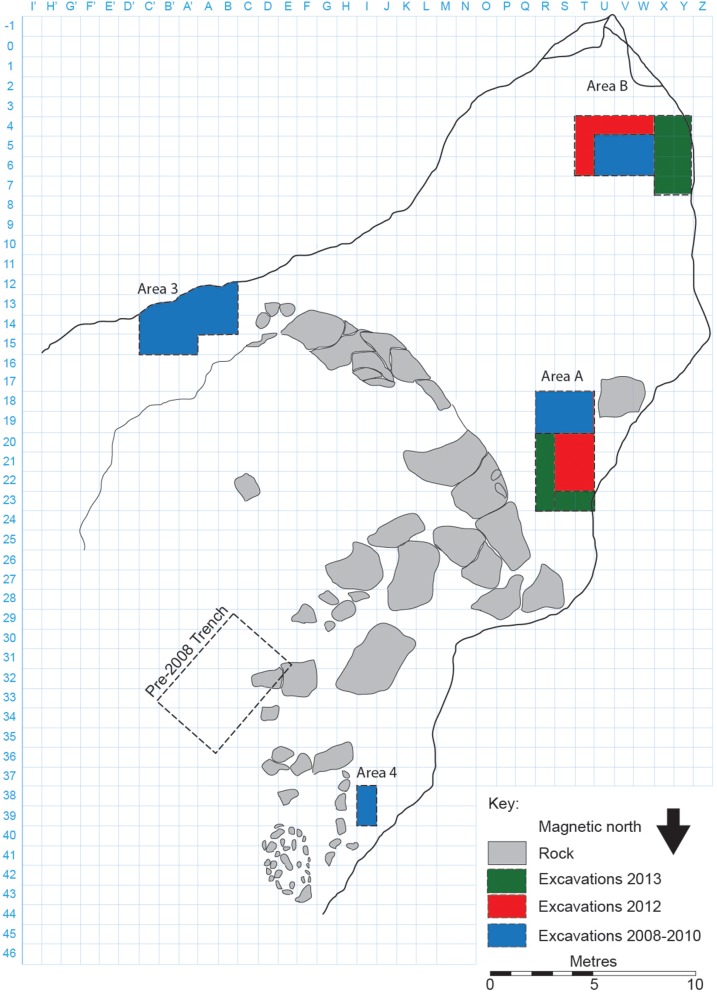
Plan of Satsurblia Cave.

The 2012–2013 excavations in Satsurblia were conducted in two areas. Area A is situated in the north-western part of the cave, near the entrance (squares R−T 20−24). Area B is in the rear of the cave (squares T−Z 4−7), adjacent to a trench excavated by K. Kalandadze in the early 1990s. Both areas revealed stratigraphic sequences comprising Pleistocene (Upper Palaeolithic) and Holocene (Eneolithic and more recent) deposits (Fig. 2).

### Area A

**Figure 3:**
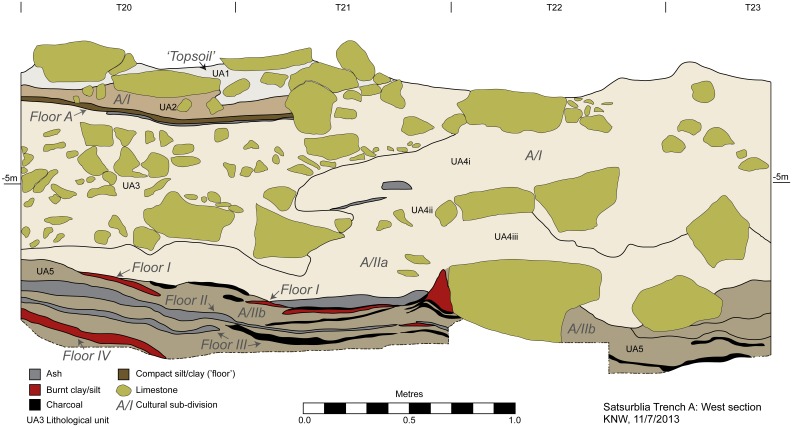
Transverse section of Area A, west.

The exposed stratigraphic sequence of the Area A so far includes five main lithological units (labelled A1–A5, with additional subdivisions, e.g. A4i–iii) corresponding to three main archaeological strata (labelled A/I, A/IIa, A/IIb) (Fig. 3). All the observed layers in Area A are associated with an extremely large boulder that is situated to the south of the area (in squares R-T 17−19), the upper face of which is exposed on the current surface of the cave, continuing to an as yet unexcavated depth (observed height of 1.5 meters).

Under the ‘topsoil’ (Unit A1), representing incipient pedogenesis within the upper levels of the underlying units, the deposits are formed mostly of open, matrix-supported pebble, cobble and boulder gravels, with occasional lenses of well-sorted silts and clays. This series of units (A2−A4ii) are grouped as the archaeological layer A/I and represent multiple episodes of sedimentation, occupation and roof collapse in the cave during the course of the Holocene.

Archaeological layer A/I yielded moderate quantities of finds – Eneolithic and (in the upper parts) Classical and Medieval pottery, as well as lithic items which seem mostly to derive from the earlier, Upper Palaeolithic levels. At least one discrete occupational event might be identified within Layer A/I (at an elevation of 4.30−4.40 m below the datum): a burnt, nearly horizontal floor-like surface consisting of yellowish brown clay with frequent granular to coarse sand-sized charcoal fragments (“Floor A”). Although there is a scarcity of cultural material directly associated with this feature, it is nevertheless cautiously interpreted as being of Eneolithic date.

The total thickness of the A/I layer varies widely: in the northwestern part of the excavated area (squares T22−23) it is ca. 80 cm (4.00 v4.80 m below the datum), while in the east (squares R20−23) it is ca. 40 cm (4.00−4.40 m below the datum). Consequently, the upper parts of the underlying Upper Palaeolithic Layer A/II (namely A/IIa), which are well preserved in the eastern part of the area, are truncated by erosion. In the northernmost part of the area (squares R-T24), a large depression with vertical walls (an excavated pit?) filled in with boulder-pebble-sized clasts is associated with Layer A/I. Its depth is at least 60 cm (4.90−5.45 m below the datum) and it continues to a yet unknown depth. A series flat boulder-sized horizontally laid stones (more than 1 m in diameter) is observed in the lower part of the pit. The feature contains Eneolithic pottery.

**Figure 4:**
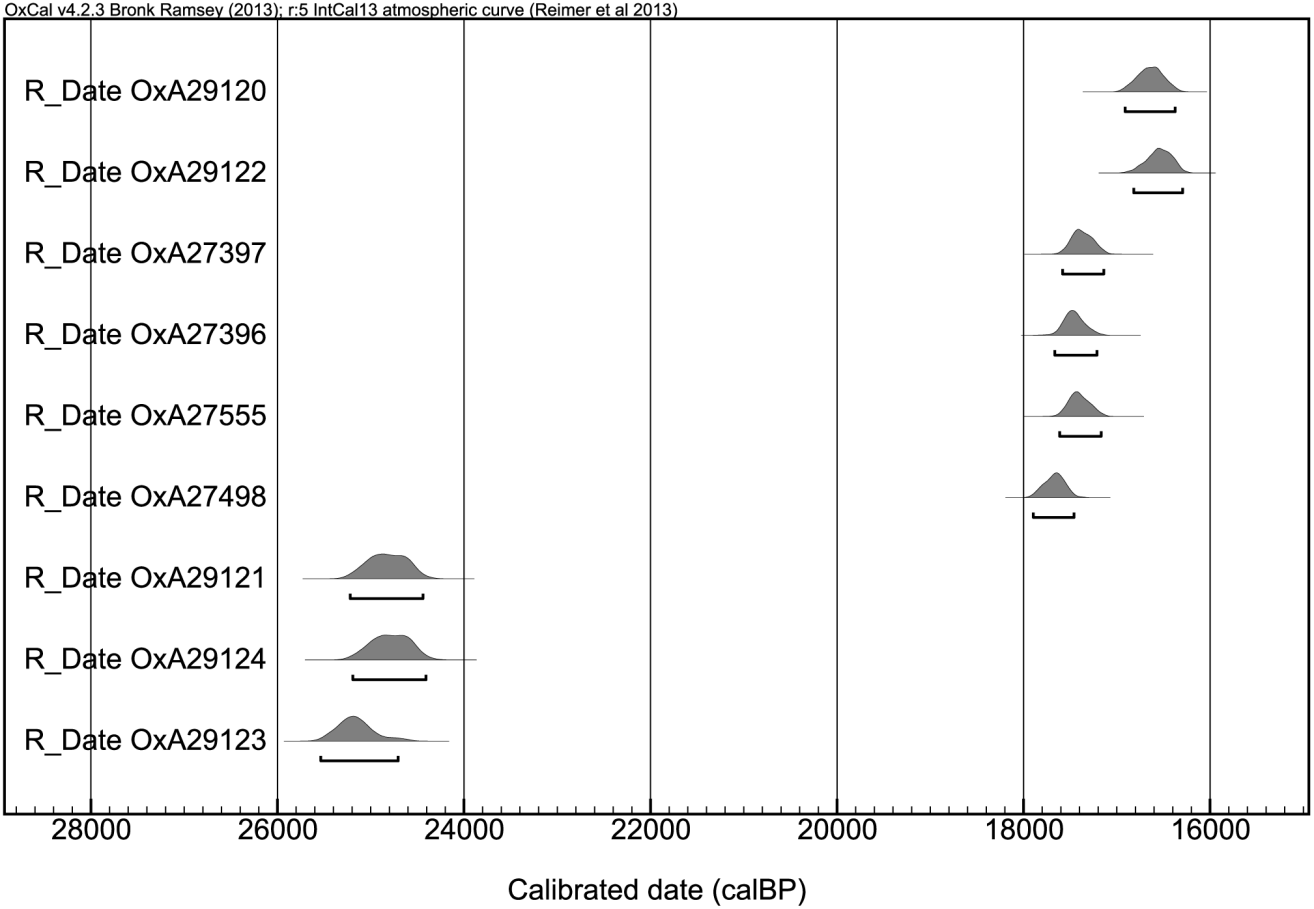
Absolute chronology of Units A/iia, A/IIb, B/II and B/III based on calibrated radiocarbon determinations.

The Upper Palaeolithic archaeological layer (A/II) is associated with lithological units A4iii and A5. In the western section of the area, the upper part of these strata (lithological unit A4iii/Archaeological layer A/IIa) comprises a brown fine cave earth of silt (mostly) and clay with moderate quantities of granular limestone clasts, occasional sub-angular limestone pebbles and granular-fine pebble-sized charcoal. Localized combustion features are manifested by a red coloration of the sediments. The layer is dated to 16,911−16,215 cal. BP (95.4% confidence interval, Fig. 4, Table S1).

Lithological unit A5 (archaeological layer A/IIb) comprises a complex of anthropogenic deposits including at least four discrete occupation episodes (Floors 1–4 identified in T-S 23−20), represented by compact surfaces of silt/clay. In the eastern part of the area (R23−20), where the upper part of the Upper Palaeolithic stratigraphy had not been truncated by later erosion, the occupational surfaces with burnt material are also observed higher up, in the AII/a sub-layer. By the end of the 2013 season two of those surfaces (labelled “Floors B, and C”) had been excavated. Most probably the lowermost of those surfaces correlate with Floors 1−3 of the western section (Layer A/IIb). The layer is dated to 17,895–17,140 cal. BP (95.4% confidence interval, Fig. 4, Table S1). The layer does not chronologically overlap, even at 95% confidence with dates obtained for A/IIa, and hence lends support to the hypothesis that the layers contain evidence of separate occupations. Nevertheless, there is no observed stratigraphic unconformity between the layers and hence it is plausible that future fieldwork and further dating might point to a continuity of occupation during the period 17.9–16.2 ka cal. BP.

The topography of the excavated ‘floors’ is generally irregular, with numerous shallow depressions, typically filled with charcoal, burnt pebbles, flints and bones. In one case (Floor 2, Layer A/IIb) a circular hearth constrained by medium-sized cobbles and covered by a thick layer of charcoal and ashes was identified in square T22.

Micromorphological analysis was carried out on (1) two block samples from the fireplace hearth in Floor 2, Layer A/IIb, square T22d, and (2) two block samples from Floor 1, Layer A/IIb, T23b. Information about sample preparation is provided in Text S1. Most sedimentary components are identical in all samples. The coarse components (those larger than silt, >62 µm), include sand-sized to medium pebbles (cm-sized) angular to sub-angular clasts of limestone, which are relatively common and likely derived from the cave walls and ceiling (*éboulis*) as well as occasional angular fragments of chipped fine-grained siliceous rock which are likely debitage from stone-tool production. Sand-sized fragments of burnt bone and finely comminuted fragments of charcoal and humified wood are dispersed evenly throughout the samples. Occasional rounded grains, 0.5 mm in size, of secondary carbonate, and with the appearance of “sinter” were also detected. Sinter is a precipitate that likely formed within the cave. The rounded form of these grains suggests that they are reworked. Sand to coarse silt sized grains of quartz are also present and might indicate an aeolian component to some of the sediments.

**Figure 5:**
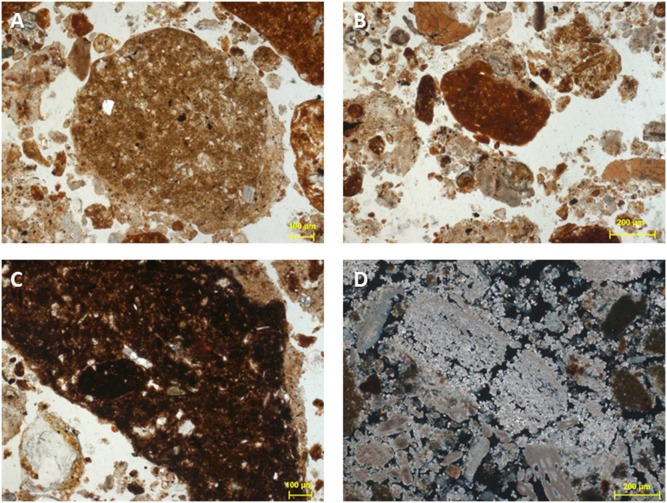
Photomicrographs of sediment thin sections from Satsurblia. At least three types of clay and silt aggregates were identified in the Satsurblia samples, based on color variation. Yellowish brown aggregates (A), reddish aggregates (B), and dark reddish brown aggregates (C). The color variation could indicate different sources, or, could be a result of differential heating of the aggregates in combustion features. The fine material at Satsurblia contains a high proportion of calcareous ash rhombs (D). A,B,C are from sample SAT-12–48 and photographed in plan polarized light (PPL). D is from sample 12–46 and photographed in cross polarized light (XPL).

Another common coarse component in the samples are rounded aggregates of reddish clay and silt-sized grains of quartz. The size of the aggregates is variable: 100 µm-2 mm. Several of the aggregates exhibit oriented clays, as evidenced by granostriated b-fabric. This characteristic is produced through rounding of the aggregates, likely during colluvial transport of the aggregates into the shelter. The aggregates display at least three different colors in Plane Polarised Light (PPL): yellowish brown, reddish, and dark reddish brown. The color difference may indicate different sources for the aggregates (Fig. 5). However, it may also indicate that the aggregates were heated in combustion features at different temperatures.

The fine fraction at Satsurblia is largely anthropogenic in the form of calcareous ashes.

A full description of the micromorphology of Floors 1 and 2, layer A/IIb, is provided in Text S1.

### Area B

**Figure 6:**
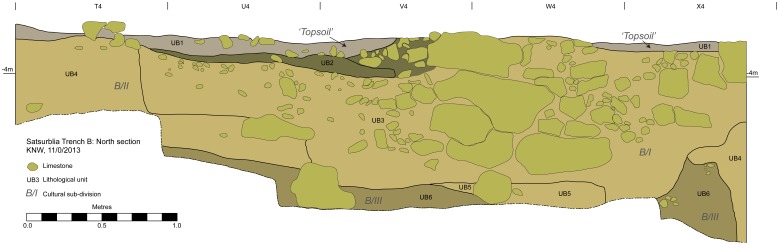
Transverse section of Area B, north.

The stratigraphic sequence of Area B includes so far six lithological units (B1−B6) comprising three main archaeological layers (B/I, B/II, B/III) (Fig. 6). In a similar way to Area A all the stratigraphic units are associated with a coarse boulder (several meters long) exposed on the surface of the cave and extending to the depth of at least 2 m below the surface. Under the thin ‘topsoil’ layer (Unit B1), a massive pit-like feature (4 m in diameter) is observed in squares U-X 4–6, and it reaches a maximum depth of 1.4 m (4.00−5.40 m below the datum in squares X4–X5). The fill of the feature (Unit B3) is a clast-supported gravel of sub-angular limestone cobbles to granules in a brown silty clay matrix. Gravel particles are chaotically distributed and are densest towards the centre of the cut feature. The material is poorly sorted and also includes numerous Eneolithic artefacts, and is most probably a deliberate archaeological fill. On the top of the fill a weathered/pedogenically worked surface (Unit B2) is observed. The pit is associated with no well-defined floor levels, is archaeologically defined as Layer B/I and dates to the Eneolithic period.

The Eneolithic pit was cut into Upper Palaeolithic layers, which are elsewhere found directly under the present-day surface (in squares T4, Y−Z 4−8). The two uppermost Pleistocene lithological units (B4 and B5), comprise a single archaeological layer, B/II, which reaches a thickness of ca. 1 m (4.15−5.25 m below the datum) and is dated to 25,220–24,440 cal. BP (95.4% confidence interval, Fig. 4, Table S1). The upper lithological unit (B4) is yellow-reddish clay containing frequent granular limestone clasts, occasional sub-angular limestone pebbles and granular to pebble-sized charcoal particles. The latter decline in frequency to the east, but form two discrete charcoal-rich fine layers in the west. Unit B4 conformably overlies unit B5, which is a brown silt/clay with moderate granular limestone clasts and which also contains frequent charcoal fragments. Its darker colour in comparison to unit B4 is a result of the presence of finely divided charcoal. Layer B/II contains a moderate quantity of Upper Palaeolithic artefacts and animal bones.

Archaeological layer B/III, associated with lithological Unit B6, has at present been excavated to the thickness of 40−50 cm (i.e. to 5.70 m below the datum). The layer is composed of a brown diamict of moderate to frequent sub-angular limestone pebbles in a silt/clay and coarse sand matrix, structured as alternate bands of clast-rich and clast-poor strata. The layer is extremely rich in archaeological finds (bones and lithics). In its upper part, in square Y6 at the elevation of 5.25–5.30 m below datum, a circular installation, 40 cm. in diameter, built of cobbles and associated with large quantities of burnt material was discovered. The layer is dated to 25,535–24,408 cal. BP (95.4% confidence interval, Fig. 4, Table S1) and hence seems to chronologically overlaps with Layer B/II. However, it is important to note that the chronology for the archaeological layers in Area B is currently based on only three radiocarbon determinations. Additional determinations will provide better information about the absolute chronology of these archaeological layers.

## Lithic Assemblages and Other Artefacts

The size and content of the lithic assemblages reflect the complexity and history of the various archaeological layers observed in the cave. In Area B the UP levels (layers B/II and B/III), were deeply cut by the Eneolithic Layer B/I and a noticeable level of mixture between the various assemblages was discerned. A similar phenomenon was observed in Area A where Layer A/I cut into the UP Layer A/II. Hence it is more acceptable to discuss for the archaeological units only general techno-typological trends. The overall picture is further distorted due to differences in samples sizes, especially for layers where samples comprise less than 100 tools (e.g., B/II with 87 items) as well as the high percentage among them of broken items (e.g., 85.4% of the tools in A/IIb), predominantly among the bladelet tools.

**Figure 7:**
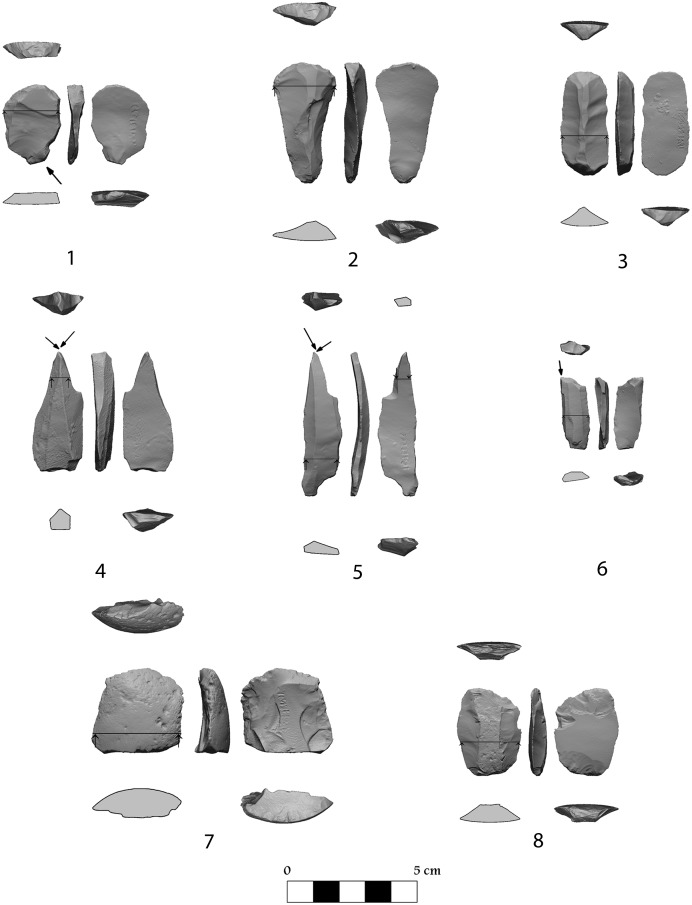
Tools from the UP layers, Satsurblia. 1–3 endscrapers; 4–6 burins; 7–8 piece esquielles.

Table 1 provides the percentages of the major tool groups in the different archaeological units. Table S2 presents the debitage counts including debris and cores. Since these are but preliminary observations, we discuss in detail only the tool types we consider as diagnostic. Still one should observe that as always in the Palaeolithic industries of the region (e.g. [12]), the endscrapers consistently account for more than 10% of the assemblages (Fig. 7∶1−3), outnumbering the burins (which vary in frequencies from 2.6% to 19.2%) (Fig. 7∶4−6), with the exception of the B/III assemblage, which points to a clear difference between the upper and lower UP entities (and see below). The same can be said concerning the *pièce esquillée* ‘tool’ type which numbers diminish from the earlier to the later assemblages (Fig. 7∶7−8). In addition to these tools the assemblages also comprise some awls and borers, notches and denticulates, retouched flakes (mostly ad-hoc) and varia.

**Table 1:** Percentages of various tool types in Satsurblia, by unit.

tool types	A/I		A/IIa		A/IIb		B/I		B/II		B/III	
	N	%	N	%	N	%	N	%	N	%	N	%
endscraper	17	14.7	60	24.5	76	11.6	40	16.8	16	18.4	17	10.9
burin	3	2.6	19	7.8	25	3.8	34	14.3	7	8.0	30	19.2
composite	0	0.0	0	0.0	3	0.5	1	0.4	2	2.3	5	3.2
blade, backed	3	2.6	7	2.9	7	1.1	3	1.3	2	2.3	0	0.0
blade, backed & trunc.	1	0.9	2	0.8	6	0.9	0	0.0	1	1.1	0	0.0
blade, truncated	0	0.0	3	1.2	8	1.2	3	1.3	1	1.1	2	1.3
blade, retouched	1	0.9	3	1.2	10	1.5	6	2.5	4	4.6	11	7.1
bladelet, backed	22	19.0	83	33.9	333	50.6	56	23.5	10	11.5	22	14.1
bladelet, backed & trunc.	6	5.2	6	2.4	36	5.5	9	3.8	5	5.7	3	1.9
bladelet, truncated	0	0.0	2	0.8	5	0.8	4	1.7	1	1.1	0	0.0
bladelet, retouched	25	21.6	18	7.3	24	3.6	33	13.9	9	10.3	38	24.4
bladelet, shouldered	1	0.9	1	0.4	7	1.1	1	0.4	0	0.0	0	0.0
flakes, truncated	0	0.0	0	0.0	1	0.2	1	0.4	0	0.0	0	0.0
flakes, retouched	2	1.7	2	0.8	2	0.3	4	1.7	3	3.4	5	3.2
fragment, backed	2	1.7	9	3.7	21	3.2	2	0.8	2	2.3	1	0.6
fragment, retouched	6	5.2	3	1.2	16	2.4	5	2.1	5	5.7	5	3.2
Gravette pt.	0	0.0	0	0.0	2	0.3	2	0.8	0	0.0	0	0.0
microgravette pt.	2	1.7	7	2.9	33	5.0	6	2.5	2	2.3	4	2.6
geometrics: lunate & triangle	1	0.9	4	1.6	1	0.2	1	0.4	1	1.1	0	0.0
rectangle	3	2.6	3	1.2	4	0.6	1	0.4	4	4.6	1	0.6
notches & denticulates	1	0.9	2	0.8	2	0.3	2	0.8	2	2.3	0	0.0
awls and borers	2	1.7	3	1.2	11	1.7	7	2.9	2	2.3	1	0.6
p. esquillee	2	1.7	0	0.0	4	0.6	2	0.8	2	2.3	4	2.6
other and varia	16	13.8	8	3.3	21	3.2	15	6.3	6	6.9	7	4.5
**Total**	**116**	**100.0**	**245**	**100.0**	**658**	**100.0**	**238**	**100.0**	**87**	**100.0**	**156**	**100.0**

The assemblages of Layer A/I and Layer B/I are quite late in the sequence (13.3−13.1 ka cal. BP) and it is clear that human occupations at those times dug into the earlier layers and consequently introduced into underlying levels some elements that are evidently intrusive, e.g., the few lunates and triangles in the A/II assemblages (and a single triangle in B/II). There are some lithics which firmly anchor A/I and B/I typologically to post Palaeolithic times: two lekalla/fishtail items in A/I [13]–[14] and a polished axe in B/I, not to mention the pottery retrieved therein (a detailed account is in preparation). When examining the lithic assemblage of A/IIa vs. A/IIb there is an observed bottom to top (A/IIb to A/IIa) decline in the percentages of microgravettes and backed and truncated items and a corresponding increase in the number of retouched bladelets (Table 1).

**Figure 8:**
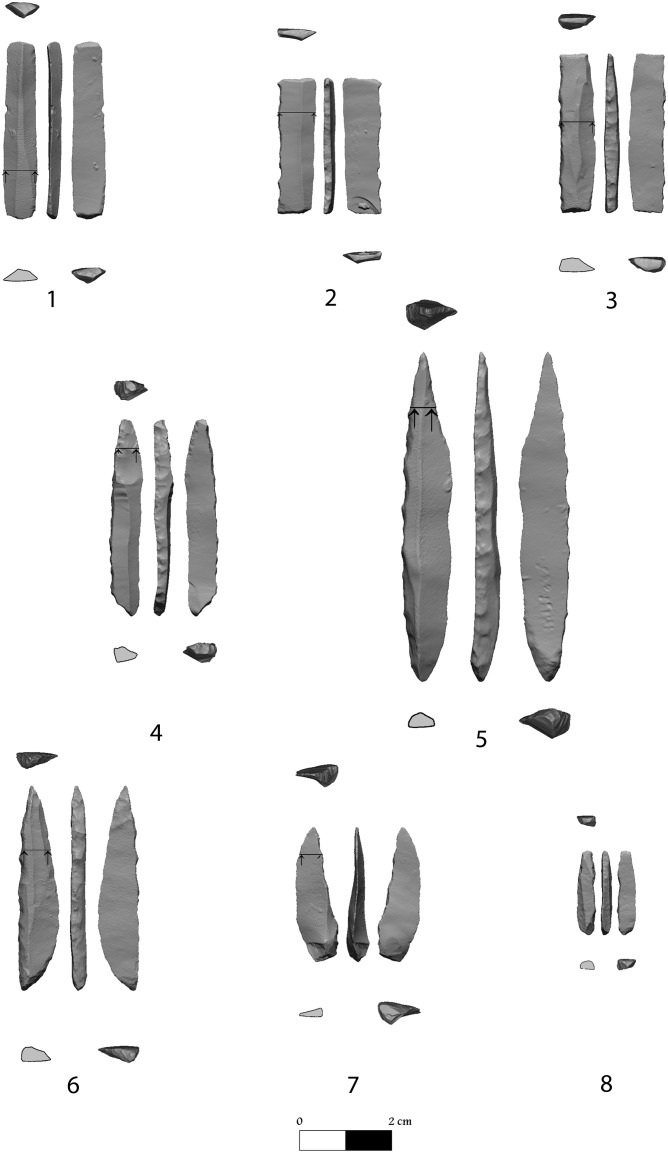
Tools from the UP layers, Satsurblia. 1–3 rectangles; 4, 6–8 microgravette varieties; 5 Gravette point.

The UP techno-typological entity best represented is one comprising microgravettes varieties (Fig. 8:4, 6−8), with rare occurrence of Gravette points (Fig. 8∶5), and some truncated varieties including rectangles (Fig. 8∶1−3) (though the numbers are low, see Table 1). It seems that these components are mostly observed in the assemblages of Layers A/IIa-b and B/II. Backed and truncated (straight) bladelets, which commonly represent broken rectangles, comprise in layer A/I –5.2%, layer A/IIa 3.3%, layers A/IIb - 6.2%, layer B/I –5.5%, layerB/II –6.9% and layer B/III - 3.2%. The frequencies of microgravette are 1.2% in A/I, 2.9% in A/IIa, 5.0% in A/IIb, 2.5% in B/I, 2.3% in B/II and 2.6% in B/III. The production technology is quite simple consisting of unipolar (the majority) and bipolar bladelet cores. It seems that most of the raw material was of poor quality, and there are many discarded core endeavours. Cores indicating intensive use are small and exhausted, indicating that the available good quality raw material was exploited to the utmost. Most of the flaked material is flint of various colours and quality with very rare obsidian pieces, including tools which comprise: in layer A/I, 3 out of 116 (3/116), in A/IIa–4/245 in A/IIb–7/658, in B/I–5/238); B/II–3/87, and in B/III–3/156. Based on the debitage analysis (Table S2) it is clear that some degree lithic production took place on site though the percentages of artefacts (debitage and tools, excluding the chips and chunks) per core greatly vary: in Layer A/I there are 680 artefacts per 12 cores (57∶1), in layer A/IIa–2391 artefacts per 26 cores (92∶1), in layer A/IIb–5316 artefacts per 12 cores (443∶1); and in Area B – B/I - 1796 artefacts per 51 cores (35∶1), in B/II–592 artefacts per 6 cores (99∶1) and in B/III–1841 artefacts per 15 cores (123∶1).

Microgravette industries, with rare Gravette points, were reported from recent excavations in the region (e.g., Dzudzuana B, [2], yet those represent a different (later) facies as they lack the other dominant component evident in Satsurblia A/II and B/II assemblages, namely the truncated items, in particular the rectangles. Another illustrative example from farther afield is the recently published assemblage from the site of Kalavan I (Armenia) which also represents a microgravette industry, but without the rectangles and the truncated items [15]. Going through the literature and material from past excavations, only the illustrations of finds from the UP layers at Gvardjilas Klde point to the existence of such a particular late UP variant in the region, similar to that from Satrusblia (and see figures in [16]–[19]). It should be emphasized that those rectangles are elongated items, different from the later Mesolithic varieties in size and retouch ([20], [21], as well as possessing ventral retouch, most probably for thinning and hafting (see Fig. 8: 4, 6, 8). Unfortunately, the site of Gvardjilas Klde was excavated in 1916 and again in 1953 by antiquated methods which preclude detailed comparisons and the material available for study comprises several assemblages lumped together (per.obs., and see the cautious statement by Golovanova et al. 2014∶215: “*Undoubtedly, the Epipaleolithic* [Late UP] *material dominates in both these not securely excavated collections*.” Still it is of interest to note that the two dates from the Gvardjilas Klde site [22], 15,960±120 (OxA 7855) and 15,010±110 (OxA 7856), uncal. BP, when calibrated, provide a calibrated interval falling between 19,560–17,955 cal. BP, (calibrated using OxCal 4.2 confidence interval of 95.4%) which hence falls in the time interval preceding human occupation in Satsurblia in Floors 2 and 3 (see Fig. 4).

It is interesting to note that the morphological difference between the rectangles and the microgravettes is expressed gradually, with many specimens exhibiting intermediate morphological characteristics. In the microgravette, in addition to the straight backed/retouched lateral edge ending in a point, there is also a truncation at the opposite end (mostly proximal but sometimes distal) which is rounded, ventrally retouched or minutely flaked that continues obliquely to the opposite lateral edge. In the rectangle the straight backed/retouched lateral edges end with two straight truncations created by regular retouch. Yet quite frequently there is also some ventral ‘treatment’ which actually makes the truncation part thinner. There are, as with the microgravettes, some intermediate types, of a truncation on one end and a rounded butt on the other and either can be inverse or dorsal. For example, there are bladelets, backed and straight, that are truncated distally with a pointed base, with inverse retouch on the opposite lateral edge. The spectrum also varies from pointed, intensely retouched Gravette points (though the latter are rare, see Table 1) to a variety in which both ends are rounded and with a ventral treatment. Indeed, basal treatment is consistent in all the tool types mentioned above. In the microgravette varieties it is at the pointed end or the base, for thinning. In the truncated items it also appears either as thinning of the base, or inversely at the truncation, or both.

The ratio of the backed *vs.* retouched bladelets (all varieties) does show a clear trend of change from dominance of simple retouch to backing. Thus the percentages in A/IIa and A/IIb show a dominance of backing vs. retouch - 36.3% *vs.* 7.3% and 51.1% *vs* 3.6% respectively. The picture differs in Area B in which the proportions of backed vs. retouched bladelets show a clear trend of a change from B/III-15.4% (backed) *vs.* 25% (retouched), through B/II–17.2% *vs.* 10.3%; to B/I–27.3% *vs*.13.9%.

Besides the lithic artefacts discussed herein, we have also recovered pottery sherds, and 41 worked bone pieces, most of them polished bone fragments and horn cores, but also some points, awls and needles, as well as an ‘incised’ item (B/III). Other ornaments include two perforated stalagmite pendants (Layer A/IIb) and a perforated and polished bovid tooth (Layer B/I−II). Of interest are small lumps and crumbs of yellow, red and brown ochre recovered from all UP layers. These finds will be further discussed in future reports.

## Palaeobotany

Palynological investigations of the archaeological deposits of Satsurblia cave have been conducted since 2007 [23]–[24]. Results show that the sediments are rich in both pollen and other organic remains of non-palynological character. Those include wood cells, spores of various fungi, microscopic remains of insects and other arthropods, and textile fibers.

More than 40 soil samples originating from various strata of the cave were analysed. The analysis has shown that climatic factors played a major part in the occupational history of the cave. Humans inhabited the cave mostly during warm and dry climatic phases. During humid and cold periods there was some standing water within the cave, at least in Area A, as is evident from remains of algae found in the samples [24].

**Figure 9:**
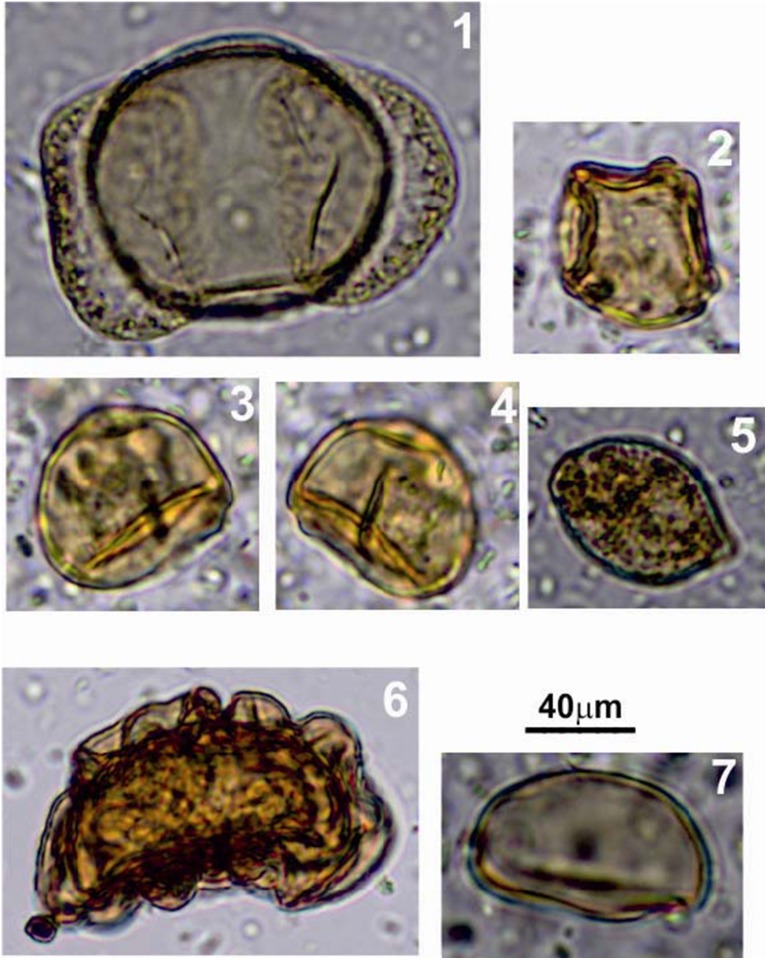
Pollen and spores, Floor 1: 1– pine (*Pinus*); 2– alder (*Alnus*); 3– hazel (*Corylus*); 5– not identified non-pollen paynomorph; 6– *Asplenium* fern; 7– *Polypodiaceae* (ferns).

A comparison of the pollen spectra of Floors 1−4 (Fig. S3) shows some variation as pollen of broad-leafed plants, including warm adapted species such as walnut is found only in Floors 1 (Fig. 9) and 2 suggesting fluctuations in the spectra of plants in the region near the cave. The pollen of Floor 1 is dominated by pine, Floor 2 by hazel, Floor 3 is very poor in pollen, while Floor 4 is also dominated by hazel. At the time of occupation of Floors 3 and 4, pollen of warm adapted plants is absent. Charred parenchyma cells of pine have been found, however, in all the four floor samples. It is possible that during formation of Floors 3 and 4 pine forest was growing in the vicinity of the cave. In the lower layers of the cave, besides the cells of pine, there were remains of pine needles [25] which, similarly to wood, cannot be transported over significant distances and therefore might serve as an useful indicator of pine forests near the cave. Moreover, the samples containing the remains of needles and pine wood cells comprise also pine pollen [24].

Additionally, large quantities of phytoliths and starch grains of cereals (Fig. S4), and the presence of Poaceae in the pollen spectra point to the importance of wild cereals in the diet of the Upper Palaeolithic inhabitants of the cave. A total of 40 flax textile fibers were recovered from all floors, including some dyed in blue and pink colours (Fig. S5).

## Microfaunal Remains

Excavated deposits of the cave contain abundant remains of small rodents, shrews, hare and bats. It is apparent from the vertical distribution of the remains that depositional rates decreased during the formation of living floors within the stratigraphic sequence, although sample size does not allow detailed consideration of fluctuations in the microfaunal assemblage within the stratigraphic layers. Initial identification of the material to general taxonomic categories at the family to genus levels is shown in Table S3 based on the molar teeth (n = 522). The data show that the assemblage is dominated by a number of species of small Arvicolinae voles and the large-bodied ciscaucasian hamster *Mesocricetus raddei*. Additional important taxa include *Apodemus* sp. wood mice, the water vole *Arvicola terrestris* and the mole vole *Ellobius* sp. Less frequent remains belong to the rodent families Sciuridae (squirrel) and Gliridae (dormice), which are likely represented by two species, the small hamster *Cricetulus migratorius* and two sub-families of shrews, Soricinae (red-toothed shrews) and Crocidurinae (white-toothed shrews). A few bat (Chiroptera) remains and a single toothless mandible and isolated incisor of the small hare *Ochotona rufesence* were recovered as well.

The taxonomic composition generally indicates affinities with both southern and northern Caucasus communities of small mammals. The modern distribution of *Mesocricetus raddei*, a dry steppe species, extends from the eastern section of the northern flanks of the Greater Caucasus into the Russian Plain, only bordering Georgia in the northeast ([25], see also [26]). Its smaller-bodied congener, *M. brandti*, occupies the southern parts of the Caucasus region into eastern Georgia. The remains of *Ellobius* sp., also a steppe species, may represent either *Ellobius talpinus*, which today is distributed north of the Greater Caucasus in the Russian Plain [27] or the southern *E. lutescens* presently found only as far north as southern Armenia [28]. More pronounced southern biogeographic affinities are indicated by the presence of rare remains of the Afghan pika *Ochotona rufesence* with a modern fragmented distribution in Iran and adjacent regions of Central Asia [29]. These specimens occur in Layers B/II. In the southern Armenian highlands this species is a dominant component of the UP preceding the LGM [30].

Present day distribution ranges of other taxa in the assemblage extend to both southern and northern sections of the Caucasus and adjacent regions. In the lower elevation (300 m asl) assemblage from Satsurblia, the steppic hamsters *Mesocricetus* and *Cricetulus* and other Arvicolinae are not as dominant as in Middle Palaeolithic layers at Kudaro Cave (1,600 m asl) [31]. The absence of *Ellobius* at Kudaro Cave may be due to its much higher elevation although this taxon was present at an even higher elevation of 2,040 m asl at Hovk Cave in northern Armenia in upper layers dating between the early Upper Palaeolithic and the Holocene [32].

**Figure 10:**
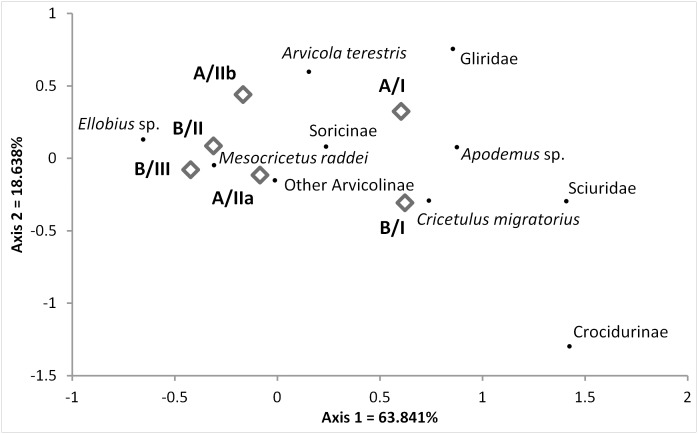
Correspondence analysis of abundances of micromammalian taxa in different stratigraphic units based on NISP data from molar teeth shown in Table S3.

The composition of taxa is fairly consistent along the stratigraphic units with an absence of rare species in some of the layers, likely due to sample size variation. This indicates that taphonomic mechanisms, mainly predation, were similar throughout the sequence. However, detailed taphonomic analysis will be needed to establish this claim. Variation can be detected in the taxonomic abundances which are analyzed using correspondence analysis in Fig. 10. The units are clearly organized along a horizontal gradient (Axis 1 = 63.84%) from an emphasis on steppic species on the left side to forest or woodland species (*Apodemus* sp., Sciuridae, Gliridae and Crocidurinae) on the right. Steppic and likely colder and more arid conditions are indicated for Layers B/II and A/IIa-b. Layers A/I and B/I, which are younger (tentatively assigned to Terminal Pleistocene based on the above-mentioned data), fall on the right side of the plot indicating a more forested environment. Nonetheless, forest taxa do not become overly abundant even in these later layers (ca. 31% of molars in each layer) and Terminal Pleistocene forests may have been less dense than the present humid deciduous forest of western Georgia.

## Macro-Faunal Remains

**Figure 11:**
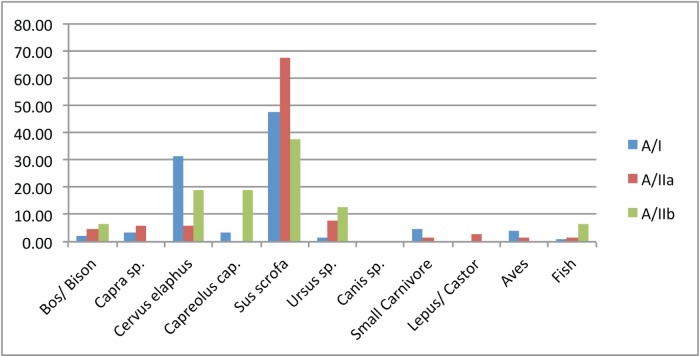
Faunal spectrum of identified species for UP Layers A/I, A/II (A/IIa and A/IIb).

The large faunal assemblage from Area A comprises a total of 327 complete and fragmentary bone specimens that were identified to taxon (excluding elements that were identified only to body-size group). Of those, 246 specimens were associated with Layer A/I, 65 specimens Layer A/IIa and 16 specimens with Layer A/IIb (Fig. 11). The taxonomic composition of layers A/I and A/II is similar and dominated by large ungulates, mainly boar (Sus *scrofa*) and red deer (*Cervus elaphus*) which comprises more than half of the identified elements in these assemblages. Other ungulates represented include large bovids (*Bos primigenius* and/or *Bison priscus*), tur (*Capra caucasica*) and roe deer (*Capreolus capreolus*).

In Area B the UP assemblage (Layer B/II) consists of only 37 bones that were identified to taxon, of these were mainly of red deer, and boar as well as two bovid bones five capra bones and one bone of *Ursus* sp.

Carnivores are represented mainly by the remains of brown bear (*Ursus arctos*; identification was based on morphological and size criteria of selected bones following [33]–[35]. Additionally, there are remains of wolf (*Canis* sp.) and small carnivores, including fox (*Vulpes vulpes*). None of the carnivore bones was found in articulation and their remains were randomly dispersed across the Upper Palaeolithic horizontal strata and were mixed with the remains of the ungulate taxa.

The Upper Palaeolithic bone assemblage includes also a few bones of medium-sized species and two fish vertebrae. Additionally, we report the remains of Eurasian beaver in Layers A/I and A/IIa (*Castor fiber*). This species was once widespread in the Caucasus [36]–[37] until local extinction at the end of the 19th century [38].

The boar remains are dominated by a high number of young individuals (at least 25% are under the age of 24 months; based on DP_4_/M_3_ ratio following [39]. In addition we note the presence of two neonate specimens at age of less than three months (based on [40]). Assuming that wild boar in the Caucasus give birth to their young in early spring (in March–April, according to Heptner et al. [41]), the remains represent animals killed during late spring–early summer. The red deer and tur remains are dominated by prime-aged individuals. Also the bear remains derived from a prime-age individual.

The bone assemblage exhibits excellent preservation as evidenced by the presence of a whole range of bone densities, including porous parts such as sternum fragments. Bone preservation does not seem to vary among taxa. The long bones showed minor signs of surface weathering, indicating rapid burial of finds and the cave’s protective conditions. Traces of carnivore bone ravaging activities are few (n = 9) and are present on the remains of all ungulate taxa. Rodent gnaw marks are also present in low numbers (n = 3). It appears that scavenging animals had only secondary access to the food remains.

Preliminary analysis of breakage patterns and bone surface modification reveals that the dominant agents of bone accumulation and bone damage were the humans. Virtually all ungulate long bones were split open to obtain marrow, evident by the high ratio of fresh (green) fractures (over 80%, and following the typology of Villa and Mahieu, 1991 typology [42]). Butchery marks are observed on boar and cervid, representing all butchery and carcass processing stages (skinning, dismemberment, and filleting).

## Discussion

During the past two decades renewed and new excavations of cave sites in western Georgia have placed a major focus on the timing and nature of the Middle-Upper Palaeolithic transition, Neanderthal extinction and the arrival of modern humans to this region [1]–[3], [11], [13].

Many of the excavated cave sites in Western Georgia, such as Ortvale Klde, Sakajia, and Ortvala did not provide post-LGM Upper Palaeolithic archaeological sequences. Our knowledge was therefore limited as regards human occupation in this region before, during and after the LGM. Fieldwork in Dzudzuana provided us with three well–dated UP occupational phases of which Dzudzuana C (27–24 ka cal. BP) precedes the LGM and Dzudzuana B (16.5–13.2 ka cal. BP) begins several millennia after the end of the LGM.

The Upper Palaeolithic layers in Satsurblia cave provide new information about human occupation in the southern Caucasus during the period prior to (Layers B/II and B/III) and after (Layers A/IIa-b, A/I and B/I) the LGM. Layer B/II (25.2–24.4 cal. Ka BP) chronologically overlaps with Dzudzuana Unit C, while Layers A/IIa-b, A/I and B/I have a limited overlap with Dzudzuana Unit B (16.5–13.2 ka cal BP). We can therefore report with confidence the discovery and analysis of human occupation in western Georgia during the period spanning between 17.9–16.2 ka cal. BP. The lithic analyses reveal that during this period, there existed a cultural (lithic) variant resembling the Eastern Epi-Gravettian, and similar to the industry reported from the site of Kalavan I, Armenia [15] dominated by bladelet tools, discrete among which are varieties of the microgravette point and truncated items. Moreover, besides rare occurrences of Gravette points, there is among the backed and truncated bladelets including a tool type that was not reported from earlier excavations in the region. This is the rectangle that differs from the geometric trapeze-rectangle of the proceeding Mesolithic cultures in size, shape and retouch (Fig. 8∶1–3). Additional typo-technological characteristics designate this assemblage as either a local or a temporal variant, hitherto not reported from this region.

It seems that the Gravette-microgravette-rectangles (the local Epi-Gravettian) industry identified in Satsurblia layers A/II and B/II illustrates the statement made by Bar-Yosef and co-authors [2], namely that the UP sequence reconstructed through the renewed excavations in the sites of Ortvale klde, Dzudzuana and Kotias Klde does not reflect the whole picture, and that we are still missing diachronic and synchronic techno-typological varieties which most probably existed in the region. It appears that the excavations at Satsurblia are providing some of the data to fill in those gaps in the Georgian UP sequence. Moreover, based on the nature of B/III assemblage we can cautiously state that the B/III assemblage represents a pre-Gravette/microgravette stage (in accord with the available dates) though we still cannot define its techno-typological characteristics in detail. Future excavations will both enlarge the samples of the assemblages discussed above as well as provide material from earlier times as we go deeper unto the occupation layers observed on site.

Another unique feature is the presence of ‘floors’ that are formed almost entirely as a result of anthropogenic processes. This is indicated by the micromorphological studies which point to the presence of reworked ashes and deposits, seemingly a result of hearth cleaning activities. This is further supported by the analyses of starch grains of wild grasses and the relatively high quantities of Flax fibers which were obtained from the 4 samples taken from Floors 1–4 (Layer A/IIb). These living surfaces therefore provide the opportunity to examine for the first time in the southern Caucasus, the study of intra-site spatial variation before and after the LGM.

**Figure 12:**
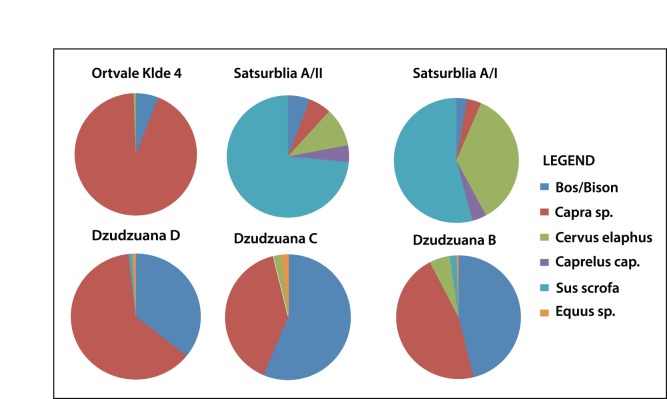
Regional pie charts of the main ungulate species in excavated UP assemblages.

The macro-faunal assemblage represents repeated seasonal visits by UP hunters targeting mainly forest-dwelling ungulates. *Sus scrofa* dominates in both Levels A/I and A/II (61.7% and 47.6%, respectively). The abundance of *Cervus elaphus* in Level A/II is 8.6% increasing to 31.3% in Level A/I. The common presence of prime-age prey implies deliberate hunting. In addition the remains of juvenile boar indicate that at least some of the hunting episodes took place during late spring - early summer. A similar pattern was observed for the assemblage from Layer B/II which is dominated by red deer and boar (73%–27/37 bones). Other UP and Early Mesolithic archaeo-faunal assemblages from the region are often dominated by open-landscape taxa (*Capra caucasica* and Bos/Bison in UP Dzudzuana Cave, both pre-LGM (Unit C and D) and post-LGM (Unit B) assemblages, and MP-UP Ortvale Klde; [9]–[11] (Fig. 12). The assemblage from the open-air UP site of Kalavan 1 in northern Armenia (dated to ca. 18–16 ka cal. BP [15] mainly consists of remains of open-landscape wild Caprinae (Ovis sp./Capra sp.). In all of the above-mentioned sites except Satsurblia there is a predominance of a single taxon suggesting a focus on specialised hunting, a pattern that differs from the one we report here for Satsurblia in which the faunal assemblage consists of a more diverse array of woodland dwelling ungulates. A somewhat similar pattern and composition of taxa was identified in Mesolithic Kotias Klde [43].

The different patterns of species abundances of hunted game when comparing Satsurblia to the above-mentioned Georgian UP sites reflect differences in the geographic location of the sites in relation to climate and vegetation zones. Satsurblia is located in a lower elevation part of western Georgia, well within the present day humid deciduous forest zone influenced from the Black Sea climatic regime. In contrast, the sites of Dzudzuana, Ortvale Klde and Bondi Caves are located 50 km east of Satsurblia, at a higher elevation (∼600–800 asl) region of Chiatura, in the foothills of the Greater Caucasus Mountains [2], [4]. The dominance of open-landscape taxa at these sites indicates either a preference of UP hunters for utilizing open landscapes combined with better access to such areas or a limited easterly shift of the climatic zones between the UP and present day.

Both the micro- and macro-faunas indicate a more dense distribution of woodland ecosystems associated with a climatic amelioration during the post-LGM human occupation of Satsurblia (i.e. between ca. 18–13 ka cal. BP). The microfaunal component overall points to an increase in woodland habitat when comparing the pattern in the pre-LGM (layers B/II and B/III) and post-LGM phases (layers A/IIa-b). This is supported by macrofaunal evidence for an increase in *Cervus elaphus* frequencies in the upper levels (AII-AI) and the persistent presence of pollen and wood cells of arboreal vegetation including *Pinus*, *Juglans* and *Corylus* throughout Layer AIIb. The existence of wetlands in the vicinity of the site is indicated by the presence of the Eurasian beaver, a key indicator species of wetland ecosystems, and the rodent water vole which occurs throughout the sequence (*Arvicola* sp.). A similar gradual forestation trend through post-LGM times and the final Pleistocene was recently detected in studies of micromammalian faunas in the Iberian Peninsula [44] and southern Italy [45]. It is suggested that in spite of the major biogeographic barrier of the Greater Caucasus Mountain Range, the region as a whole was permeable to biotic exchanges in different directions in relation to changing distributions of vegetation zones and climatic shifts up until relatively recent times of the final Pleistocene. Relative simplification of the vegetation cover during cold phases such as the LGM and subsequent Heinrich 1 event may have aided the movements of specific biotic elements, although not of entire communities, across steep mountainous sections of the Caucasus and provided the background for shifting patterns of human occupation of the region.

## Conclusions

The study of the UP Layer in Satsurblia has revealed evidence of human occupation during the pre-LGM period (Area B, Layers B/II and B/III), 25.5–24.4 ka cal. BP, an interval broadly contemporaneous with part of the occupation in Dzudzuana C (27–24 ka cal. BP). The chronology of Layer AII/a and A/IIb indicate the presence of a new post-LGM phase (Area A, Layers A/IIa and A/IIb: 17.9–16.2 ka cal. BP). The latter provides new evidence for human occupation in this region more than a millennium prior to what was previously known based on the radiocarbon-based chronology of Dzudzuana B (16.5–13.2 ka cal. BP, [2].

The lithic analyses reveal that during this post-LGM period, there existed a cultural (lithic) variant of the Eastern Epi-Gravettian, dominated by bladelet tools, discrete among which are varieties of the microgravette point. While microgravette industries, with rare Gravette points were reported from Dzudzuana B [2], those represent a later facies as they lack the other dominant component evident in Satsurblia A/II and B/II assemblages, namely the truncated items, and in particular the rectangles which were not reported from earlier excavations in the region.

The recovery of living floors and the presence of combustion features and hearths provide new information about the processing of wild cereals, the utilization of flax, and wood, as well as paleoenvironmental reconstruction based on palynological and micromorphological analyses. These indicate that during the formations of Floors 1–4 Layer A/IIb, the environment in the vicinity of the cave was predominantly temperate coniferous forest but with some evidence for pollen of deciduous taxa. Both the micro- and macro-faunas point to a relatively dense distribution of a wooded ecosystem during the post-LGM human occupation of Satsurblia (18–13 ka cal. BP).

The macrofaunal analysis indicates that subsistence focused on the hunting of wild boar and red deer as well as some wild goats and wild bovines. The Satsurblia UP assemblages differ from those reported for other sites with UP phases in the region in which hunting focused on bos/bison or wild goats. Future research will clarify whether these difference in the composition of UP macrofaunal assemblages from sites in this region reflect for the most part variations in the availability of animal resources by season and period [2], [9], [11], or also implies variations which are associated with cultural preferences.

The results of the campaigns in Satsurblia and Dzudzuana suggest that at present the most plausible scenario is one of a hiatus in the occupation of this region during the LGM (between 24.4–17.9 ka cal. BP). Future fieldwork will aim to assess earlier occupations and in particular to investigate whether the hiatus in occupation in Dzudzuana between Units D (34.5–32.2 ka cal. NP) and C (27−24 ka cal. BP) also occurs in Satsurblia, suggesting an additional regional (and potentially pan-regional) occupational hiatus.

## Materials and Methods

### Ethics

All necessary permits for the Satsurblia fieldwork and analyses of all sediments, human remains and artefacts, were obtained by Tengiz Meshveliani (Director of the excavation) and David Lordkipanidze (Direector Genral, Georgian Nationl Museum).

## Supporting Information

Figure S1 **Scan of sample SAT-12–46 T23b: Floor 1.**
(TIF)

Figure S2 **Scan of sample SAT-12–48 T22d: fireplace hearth on the 2nd floor.**
(TIF)

Figure S3 **Diagram of (a) non-pollen palynomorphs and (b) pollen and spores from samples taken from Floors 1–4 Layer A/IIb.**
(EPS)

Figure S4 **Cereal starch grain, Floor 1.**
(TIFF)

Figure S5 **Flax fiber, fireplace installation, Floor 2.**
(EPS)

Table S1 **AMS determinations from Satsurblia measured at the ORA.**
(PDF)

Table S2 **Debitage counts by Layer, including debris and cores.**
(XLSX)

Table S3 **Abundances of micromammalian taxa as numbers of identified specimens (NISP) and minimum numbers of individuals (MNI) by stratigraphic units and elevation within the stratigraphic sequence.**
(XLSX)

Text S1 **Supporting information about the micromorphological analysis.**
(DOCX)
