# Supplementary figures and images for: Satsurblia: New Insights of Human Response and Survival across the Last Glacial Maximum in the Southern Caucasus
Source: PLoS One. 2014 Oct 29;9(10):e111271. doi: 10.1371/journal.pone.0111271 (PMC4213019; doi:10.1371/journal.pone.0111271)

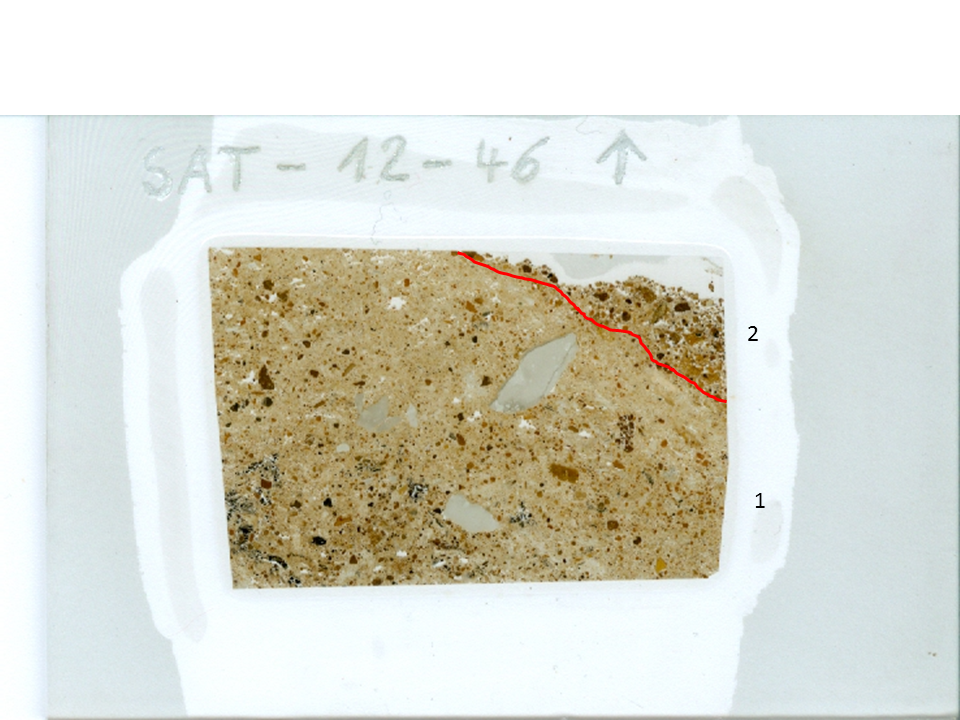

Supplement: Figure S1 — Scan of sample SAT-12–46 T23b: Floor 1. (TIF) [file pone.0111271.s001.tif]

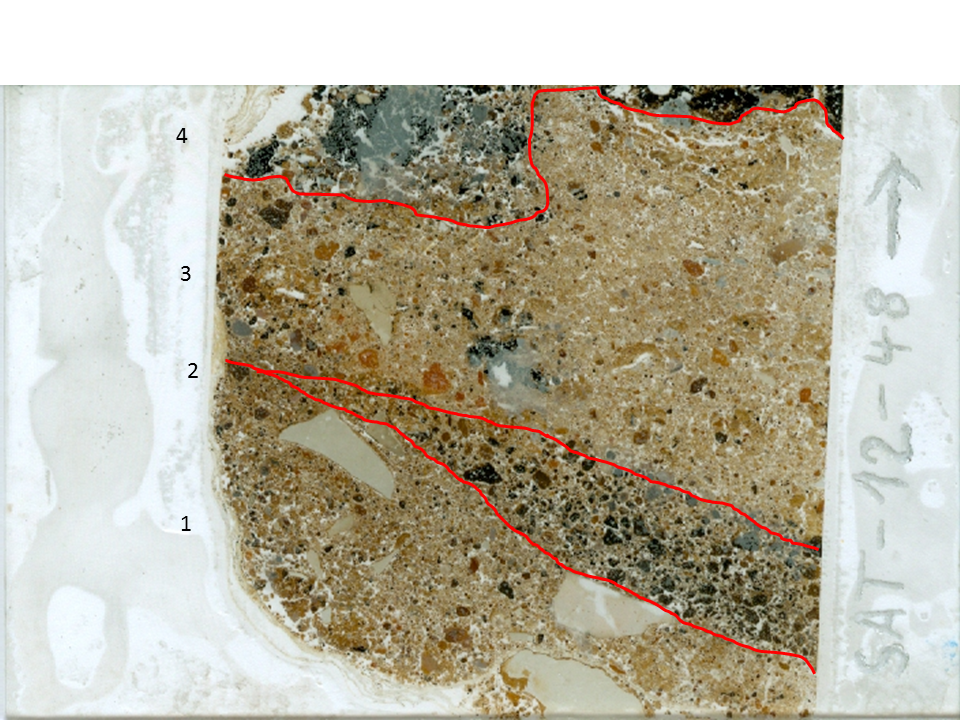

Supplement: Figure S2 — Scan of sample SAT-12–48 T22d: fireplace hearth on the 2nd floor. (TIF) [file pone.0111271.s002.tif]

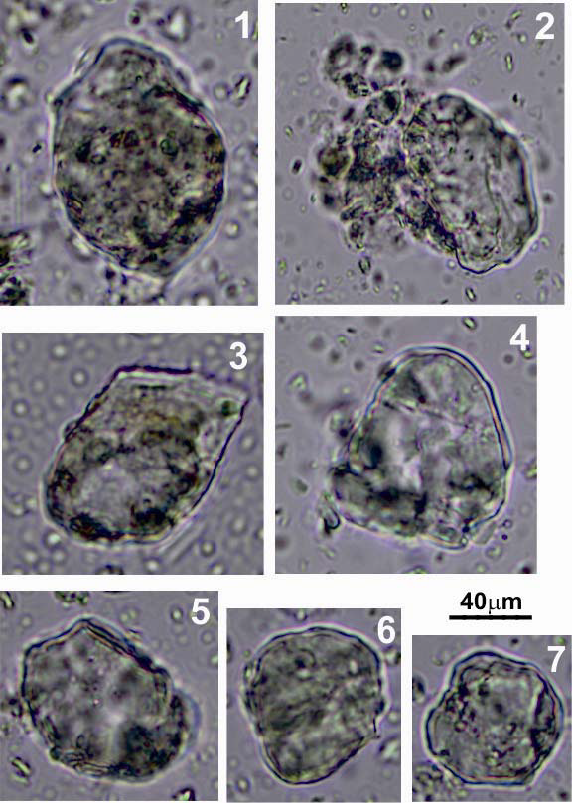

Supplement: Figure S4 — Cereal starch grain, Floor 1. (TIFF) [file pone.0111271.s004.tiff]
