# Supplementary material for: Satsurblia: New Insights of Human Response and Survival across the Last Glacial Maximum in the Southern Caucasus
Source: PLoS One. 2014 Oct 29;9(10):e111271. doi: 10.1371/journal.pone.0111271 (PMC4213019; doi:10.1371/journal.pone.0111271)
Supplement: Table S1 — AMS determinations from Satsurblia measured at the ORA. (PDF) [file pone.0111271.s006.pdf]

Table S1. AMS determinations from Satsurblia measured at the ORAU, all on ungulate bones

Layer	OxA	Date	+/-	Weight	% Yield	%C	$\delta^{13}C$ (‰)	C:N
**Area B**								
B/III	29123	20,860	120	600	3.7	42.8	-19.2	3.4
B/III	29124	20,580	120	600	3.3	43.7	-19.2	3.3
B/II	29121	20,610	120	610	3.2	44.2	-18.3	3.3
**Area A**								
A/III	27498	14,490	70	650	1.2	46.7	-19.1	3.3
A/III	27555	14,290	65	640	2.2	43.3	-18.4	3.2
A/III	27396*	14,330	65	940	3.7	41.8	-19.0	3.2
A/III	27397*	14,265	65	690	2.1	44.1	-18.9	3.1
A/II	29122	13,705	60	610	9.6	44.7	-18.7	3.4
A/II	29120	13,765	60	600	4.9	44.3	-18.3	3.2

*Duplicate samples.

All are ultrafiltered gelatin samples. Stable isotope ratios are expressed in ‰ relative to Vienna Pee-Dee Belemnite. Mass spectrometric precision is ±0.2‰ for carbon. Weight used is the amount of bone pretreated, and the yield represents the weight of gelatin or ultrafiltered gelatin in milligrams. %yield is the wt% collagen, which should not be <1 wt% at the ORAU. This is the amount of collagen extracted as a percentage of the starting weight. %C is the carbon present in the combusted gelatin. For ultrafiltered gelatin this averages 41.0 ± 2%. C:N is the atomic ratio of carbon to nitrogen. At the ORAU this is acceptable if it ranges between 2.9 and 3.5.
