# Supplementary material for: Satsurblia: New Insights of Human Response and Survival across the Last Glacial Maximum in the Southern Caucasus
Source: PLoS One. 2014 Oct 29;9(10):e111271. doi: 10.1371/journal.pone.0111271 (PMC4213019; doi:10.1371/journal.pone.0111271)
Supplement: Text S1 — Supporting information about the micromorphological analysis. (DOCX) [file pone.0111271.s009.docx]

**Satsurblia: new insights about human response and survival across the Last Glacial Maximum in the southern Caucasus.**

**Supporting Information**

**Text S1.**

**Micromorphological analysis**

**a. Sample preparation:**

Upon receiving, the samples were oven-dried for ca. 24 hours at 60°C. They were then indurated under vacuum with a mixture of polyester resin, styrene, and Methylethylketone peroxide. After approximately 2 weeks, when the resin had achieved a gel-like consistency, the samples were heated again for ca. 24 hours at 60°C to completely harden them. We sliced the samples with a rock saw and produced thin sections from these slices in the Micromorphology Laboratory at the University of Tübingen. Thin sections are 60x90mm in dimension and 30µm thick. The thin sections were analyzed in the Geoarchaeology Laboratory at the University of Tübingen using a Zeiss Axioimager petrographic microscope with magnification between 25-200x, under plane polarized light (PPL), cross polarized light (XPL) and blue light fluorescence. Descriptions and terminology follow standard procedures described by Stoops [1] and Courty et al. [2]

**b. Micromorphology of Floors 1 and 2**

**b.1 Micromorphology of Floor 1 (sample SAT-12-46, Fig. S1):** the sample consists of two distinct units. The lower unit (unit 1) contains the typical sedimentary components at Satsurblia. It exhibits a granular to lenticular microstructure. Some zones of lenticular microstructure appear in situ (i.e., not macro-aggregates), but others seem to be macroaggregates. The ashes here appear “fresher” than in other samples: they are not recrystallized and do not exhibit much iron staining. Some thin, laminated ashes are present. These may indicate evidence for in situ combustion. However, these laminated ashes have been disturbed by the formation of a lenticular microstructure which makes it difficult to say with certainty if the features are in situ or reworked. Unit 2 has a higher concentration of rounded clay/slit aggregates. Sub-angular macro-aggregates of partially cemented ashes with rounded clay aggregates are present.

**b.2 Micromorphology of Floor 2 (sample SAT-12-48, Fig. S2):** Four distinct units were identified in the fireplace sample. The lowermost (unit 1) contains the typical components described above. The sedimentary components exhibit a granular to micro-granular microstructure, which is typically formed by bioturbation, although cryoturbation can sometimes produce a similar-looking microstructure. Many of the coarse components exhibit cappings, which indicates that fine sediment (here, ashes) have been translocated through the profile. Unit 2 appears generally similar to unit 1; however, it contains a much higher proportion of rounded clay which is almost completely dominated by the “dark reddish brown” type. Most of these aggregates are between 1-2mm in size and rounded to sub- rounded. The unit exhibits a granular microstructure. The contact to unit 1 is sharp and the upper contact to unit 3 is relatively diffuse. Unit 3 has a similar composition to units 1 and 2, but with fewer clay/slit aggregates compared to unit 2. The aggregates include all varieties and no single type is dominant. The microstructure is generally micro-granular to granular. However, there are a few macro-aggregates that display vughy to lenticular microstructures. These macro-aggregates likely represent clasts or fragments of intact sediment that were not disturbed by the bioturbation that produced the granular microstructure in the rest of the unit. Lenticular microstructure is typically formed by freeze-thaw processes [3]. Unit 4 is distinct from the other units in this sample. It is composed of laminated charcoal and cemented ashes and has a massive microstructure. This unit may represent the remains of an intact combustion feature. However, the lower contact with unit 3 is sharp and irregular, and sub-angular clasts of unit 4 are incorporated into unit 3, suggesting that unit 4 has been partially disturbed by bioturbation.

**References**

1. Stoops G (2003). Guidelines for analysis and description of soil and regolith thin sections: Soil Science Society of America Inc.

2. Courty MA, Goldberg P, Macphail RI (1990) Soils and Micromorphology in Archaeology. Cambridge University Press, Cambridge, UK.

3. Van Vliet-Lanoë B (1985) Frost effects in soils. In: Boardman J, editor. Soil and Quaternary Landscape Evolution: Wiley. pp. 115–156.
